# Human monocyte-derived dendritic cells exposed to hyperthermia show a distinct gene expression profile and selective upregulation of *IGFBP6*

**DOI:** 10.18632/oncotarget.18338

**Published:** 2017-06-01

**Authors:** Arcangelo Liso, Stefano Castellani, Francesca Massenzio, Rosa Trotta, Alessandra Pucciarini, Barbara Bigerna, Pasquale De Luca, Pietro Zoppoli, Filippo Castiglione, Maria Concetta Palumbo, Fabrizio Stracci, Matteo Landriscina, Giorgina Specchia, Leon A. Bach, Massimo Conese, Brunangelo Falini

**Affiliations:** ^1^ Department of Medical and Surgical Sciences, University of Foggia, Foggia, Italy; ^2^ Institute of Haematology, University of Perugia, Perugia, Italy; ^3^ Stazione Zoologica A. Dohrn, Naples, Italy; ^4^ Dipartimento di Medicina Sperimentale e Clinica, Università degli Studi Magna Graecia, Catanzaro, Italy; ^5^ Institute for Applied Computing, National Research Council of Italy, Rome, Italy; ^6^ Department of Experimental Medicine, Section of Public Health, University of Perugia, Perugia, Italy; ^7^ Laboratory of Preclinical and Translational Research, IRCCS, Referral Cancer Center of Basilicata, Rionero in Vulture, Italy; ^8^ Institute of Hematology, University of Bari, Bari, Italy; ^9^ Department of Medicine, Alfred Hospital, Monash University, Melbourne, Australia; ^10^ Department of Endocrinology and Diabetes, Alfred Hospital, Melbourne, Australia

**Keywords:** apoptosis, B cells, chemotaxis, dendritic cells, hyperthermia, Immunology and Microbiology Section, Immune response, Immunity

## Abstract

Fever plays a role in activating innate immunity while its relevance in activating adaptive immunity is less clear. Even brief exposure to elevated temperatures significantly impacts on the immunostimulatory capacity of dendritic cells (DCs), but the consequences on immune response remain unclear. To address this issue, we analyzed the gene expression profiles of normal human monocyte-derived DCs from nine healthy adults subjected either to fever-like thermal conditions (39°C) or to normal temperature (37°C) for 180 minutes. Exposure of DCs to 39°C caused upregulation of 43 genes and downregulation of 24 genes. Functionally, the up/downregulated genes are involved in post-translational modification, protein folding, cell death and survival, and cellular movement. Notably, when compared to monocytes, DCs differentially upregulated transcription of the secreted protein IGFBP-6, not previously known to be specifically linked to hyperthermia. Exposure of DCs to 39°C induced apoptosis/necrosis and resulted in accumulation of IGFBP-6 in the conditioned medium at 48 h. IGFBP-6 may have a functional role in the hyperthermic response as it induced chemotaxis of monocytes and T lymphocytes, but not of B lymphocytes. Thus, temperature regulates complex biological DC functions that most likely contribute to their ability to induce an efficient adaptive immune response.

## INTRODUCTION

The effect of heat on cells can vary from survival and adaptation to apoptosis and, in extreme conditions, to necrosis [1-3]. These effects are either a direct consequence of heat itself on cellular components (*e.g.* denaturation of proteins) or a secondary effect mediated by cellular adaptive mechanisms such as the expression of heat shock proteins (HSPs) and the activation of selected signaling pathways. In turn, adaptive mechanisms are either related to changes in the activity of existing proteins (such as by phosphorylation/dephosphorylation or protein-protein interactions) or to changes in protein expression. Whereas the heat shock response is an ancient and highly conserved essential process for surviving severe environmental stresses, fever is a more recently evolved physiological response [4]. Importantly, since fever is also a metabolically expensive process, its phylogenetic conservation from bony fish and amphibians to mammals highlights an important role in conferring a survival advantage. In fact, it is now clear that fever is beneficial in the infected host and this depends, at least in part, upon activation of the heat shock response [5]. Although the effects of moderate heat on cellular structure and function have been extensively studied in yeast, *Drosophila melanogaster*, and tissue culture cell lines [6-8], primary human cell populations have been more rarely investigated [9,10]. This is very relevant since the effects of heat can be cell-type specific, and a variety of temperature-mediated effects on immune cell function have been reported [4,11,12].

Dendritic cells (DCs) are professional antigen-presenting cells [13-16], critical in modulating adaptive immune responses by sensing perturbations such as cell damage and inflammation in the microenvironment [17-19]. Basu et al. [20] showed that even a brief exposure to elevated temperatures has a powerful effect on the immunostimulatory capacity of DCs. Furthermore, elevated temperatures cause immature DC to mature, specifically through elevation of intracellular levels of Hsp90. Moreover, DCs exposed to thermal stress showed enhanced expression of co-stimulatory molecules (CD80, CD83, CD86) and TNF-α and improved their ability to prime autologous naïve CD8^+^ T cells [21]. Monocytes, initially described as circulating precursors for tissue macrophages, were shown by Sallusto and Lanzavecchia [22,23] to differentiate into DCs. Over the last ten years, this observation has proven to be extremely useful for the study of human DC differentiation and maturation. Moreover, *in vitro* DC differentiation from monocytes constitutes the current methodological basis for obtaining DCs for their use in DC-mediated cancer immunotherapeutic treatments [24]. In recent years, monocyte-derived DCs (moDCs) have been generated for self-vaccination of otherwise incurable tumor patient [25].

Finally, understanding novel mechanisms regulating immune response in hyperthermia can be of pivotal importance in the clinical management of patients with fever. The aim of our study was to find novel and early players involved in the febrile immune response that could possibly be exploited for diagnostic and therapeutic purposes in the future.

With these premises in mind, we investigated whether exposure to 39°C induces a distinct gene expression profile program in moDCs and whether this allows identification of genes not previously known to be part of the response to hyperthermia. We found that human moDCs exposed to hyperthermia show a distinct gene expression profile including selective upregulation of *IGFBP6.* Importantly, we show that IGFBP-6 is able to induce chemotaxis of monocytes and T lymphocytes.

## RESULTS

### DCs exposed to hyperthermia show a distinct gene expression profile

Gene expression profile analysis was performed on samples obtained from nine consecutive donor patients. In all nine cases peripheral blood CD14 monocytes were easily obtained and differentiated to DCs, and maturation to the DC phenotype could be demonstrated (Supplementary Figure S1 in Supplementary Materials) at day six, resulting in a minimum of 11.6 × 10^6^ cells ready for gene expression analysis. Incubation at 39°C for 3 and 24 h resulted in a significant increase of percentage of cells expressing maturation markers (CD11c, CD80, CD83, and HDRII), but not of CD14, if compared with cells incubated for the same duration at 37°C (Supplementary Figure S1 in Supplementary Materials).

**Figure 1:**
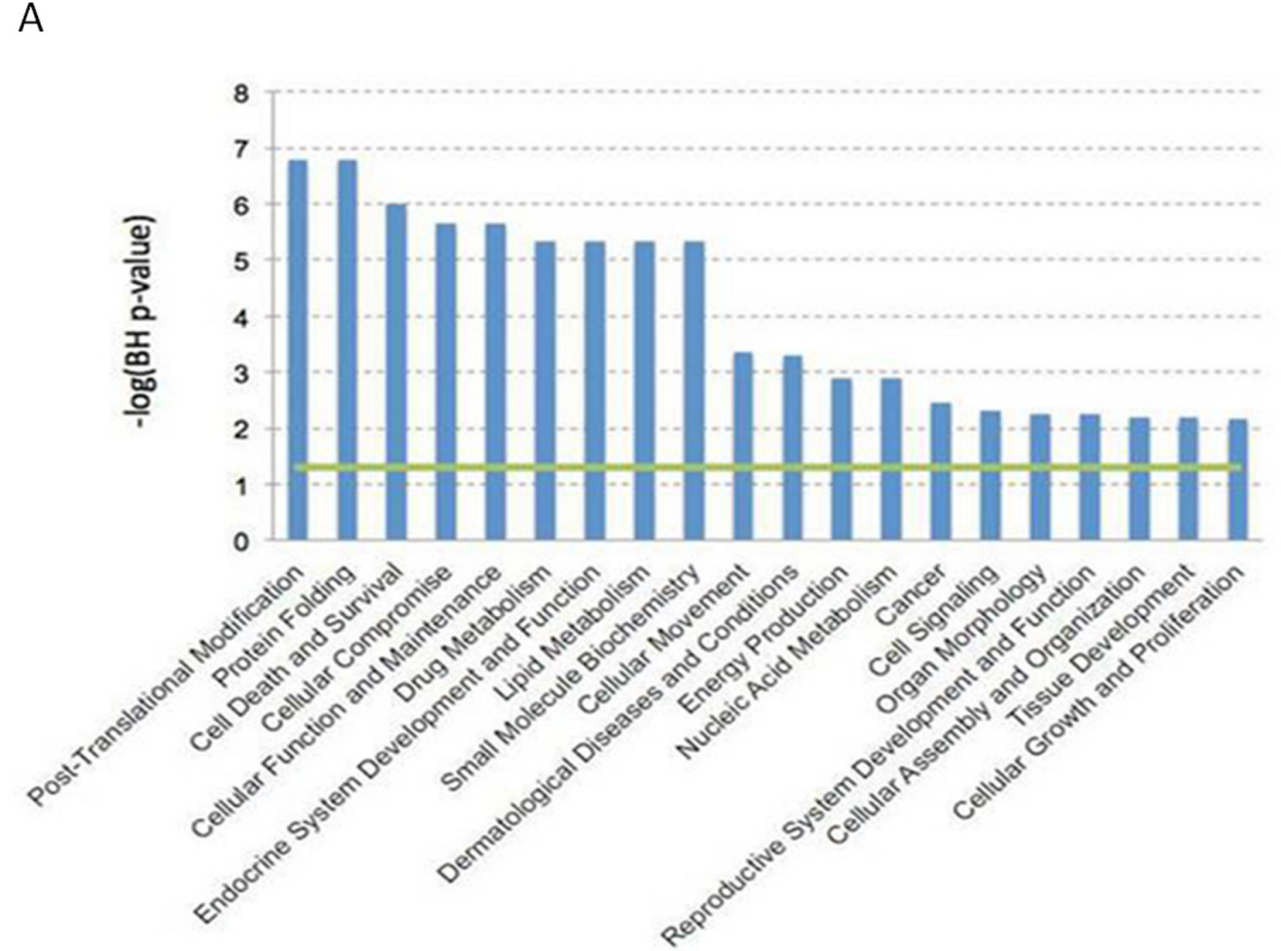
Gene expression profile data analysis Dendritic cells expression profiles were evaluated by Affimetrix HGU133 Plus 2.0 array. Data were analyzed by Expression Console (Affymetrix Inc). All the biological functions values exceeding the threshold (-log(BH *p*-value) = 1.3), represented by the green line, identified by the IPA-Biofunction tool are statistically significant (i.e. the Benjamini-Hockberg FDR corrected *p*-value < 0.05).

A 3 h-exposure of DCs to 39°C caused a significant increase in the expression of 43 genes and a decrease of another 24 genes compared with 37°C (Table 1). A biologically meaningful effect was defined as significant when a greater than 2-fold difference in expression level was detected in all experiments. Genes that underwent up/downregulation under our stress conditions belong to several different functional categories including proteins involved in post-translational modification (e.g. *NUB1* and *CACYBP*), protein folding (e.g. *CCT3*), cell death and survival (e.g. *ANXA1* and *KLF7*), and cellular movement (e.g. *IDO1*) (Figure 1). Moreover, our study points to the important role of the heat shock response in the process of DC maturation upon fever-like conditions. Notably, multiple genes encoding for heat shock proteins (*HSP90AB1*, *HSPA1A*, *HSPA1B*, *HSPA5*, *HSPA8*, *HSPB1*, *HSPD1*, *HSPE1*, *HSPH1*) were all upregulated and this finding serves also as an internal control for the experiments. These data indicate the hyperthermia may increase the maturation of monocyte-derived DCs and show that a 3 h-exposure time to hyperthermia causes a modification in the global gene expression profile of DCs.

**Table:** **Table 1.** Differentially expressed up/downregulated genes.

Downregulated		
	Gene Symbol	Fold change
228603_at	ACTR3	2.1212146
226025_at	ANKRD28	2.3790717
233011_at	ANXA1	2.3608308
225166_at	ARHGAP18	2.2429798
235088_at	C4orf46	2.0362923
229695_at	fam107b	2.348431
1554678_s_at	HNRPDL	2.129529
204334_at	KLF7	2.6032488
230636_s_at	KLF9	2.1131873
240655_at	LOC100133690	2.1164844
244846_at	MAP4K4	2.2667267
1558111_at	MBNL1	2.2078938
1569030_s_at	NUB1	2.0441904
214963_at	NUP160	2.017219
225626_at	PAG1	2.0477161
213933_at	PTGER3	2.5118344
554999_at	RASGEF1B	3.3729293
226312_at	RICTOR	2.2310023
209684_at	RIN2	2.0676558
220123_at	SLC35F5	2.102914
215078_at	SOD2	2.2981791
226837_at	SPRED1	2.4078321
207983_s_at	STAG2	2.0910532
217833_at	SYNCRIP	2.120536

Upregulated		
	Gene Symbol	Fold change
201491_at	AHSA1	3.0668783
217911_s_at	BAG3	3.3147337
219966_x_at	BANP	3.369214
228928_x_at	BANP	2.9106443
233186_s_at	BANP	2.674547
211761_s_at	CACYBP	2.099444
210691_s_at	CACYBP	2.2031457
206331_at	CALCRL	2.0365825
210815_s_at	CALCRL	2.2632575
200910_at	CCT3	2.1207094
218566_s_at	CHORDC1	4.3639574
204170_s_at	CKS2	2.359756
225434_at	DEDD2	2.2326558
225061_at	DNAJA4	5.536901
1554334_a_at	DNAJA4	4.3517003
1554333_at	DNAJA4	2.3606193
200664_s_at	DNAJB1	2.4289098
200666_s_at	DNAJB1	2.4802563
209015_s_at	DNAJB6	3.323787
213145_at	FBXL14	3.1741052
200894_s_at	FKBP4	2.268422
200895_s_at	FKBP4	2.213333
222033_s_at	FLT1	2.0301073
201503_at	G3BP1	2.117231
214359_s_at	HSP90AB1	2.6591988
200064_at	HSP90AB1	2.6207926
1557910_at	HSP90AB1	3.1781142
200799_at	HSPA1A	2.4917276
200800_s_at	HSPA1A /// HSPA1B	4.0723443
202581_at	HSPA1A /// HSPA1B	10.3153925
230031_at	HSPA5	2.2923908
211936_at	HSPA5	2.530812
201841_s_at	HSPB1	2.105686
200806_s_at	HSPD1	3.5899048
200807_s_at	HSPD1	2.923671
205133_s_at	HSPE1	3.3237238
235573_at	HSPH1	3.050632
208744_x_at	HSPH1	6.470141
206976_s_at	HSPH1	8.415396
200825_s_at	HYOU1	2.0537353
210029_at	IDO1	2.4012868
203851_at	**IGFBP6**^a^	2.2617126
207901_at	IL12B	2.0140626
227140_at	INHBA	2.0002825
202220_at	KIAA0907	3.196178
1558404_at	LOC644242	2.2391515
218559_s_at	MAFB	2.2515247
202655_at	**MANF**^a^	2.083146
217907_at	MRPL18	2.3864717
209785_s_at	PLA2G4C	2.0645976
201860_s_at	**PLAT** ^a^	2.0551631
204186_s_at	PPID	2.0264704
212706_at	RASA4	2.0581238
203164_at	SLC33A1	2.208336
212009_s_at	STIP1	2.211557
212706_at	RASA4	2.0581238
213330_s_at	STIP1	3.3498967
223330_s_at	SUGT1	2.3122559
235542_at	TET3	2.130128

^a^Genes also tested by RT-PCR are shown in bold.

### DCs selectively upregulate *IGFBP6*

Since we explored global gene expression profiling in order to search for early response genes involved in or governing the effects of hyperthermia on the immune system, it was intriguing that a few upregulated genes encoded secreted proteins, namely *MANF*, *PLAT* and *IGFBP6*, which are potentially capable of interacting with other immune cells through receptors. Mesencephalic astrocyte-derived neurotrophic factor (*MANF* or *ARMET*) encodes for a secreted protein [26] localized in the endoplasmic reticulum (ER). Reduced expression of this gene has been linked to increased susceptibility to ER stress-induced death and cell proliferation [27]. Tissue-type plasminogen activator (PLAT) is a secreted serine protease, which converts the proenzyme plasminogen to plasmin [28]. This enzyme plays a role in cell migration and tissue remodeling. Finally, insulin-like growth factor binding protein-6 (IGFBP-6) was shown to inhibit the tumorigenic properties of IGF-II-dependent cancers [29]. Also, IGFBP-6 promotes migration of Rh30 rhabdomyosarcoma cells via a distinct pathway involving an unknown receptor [30,31].

**Figure 2:**
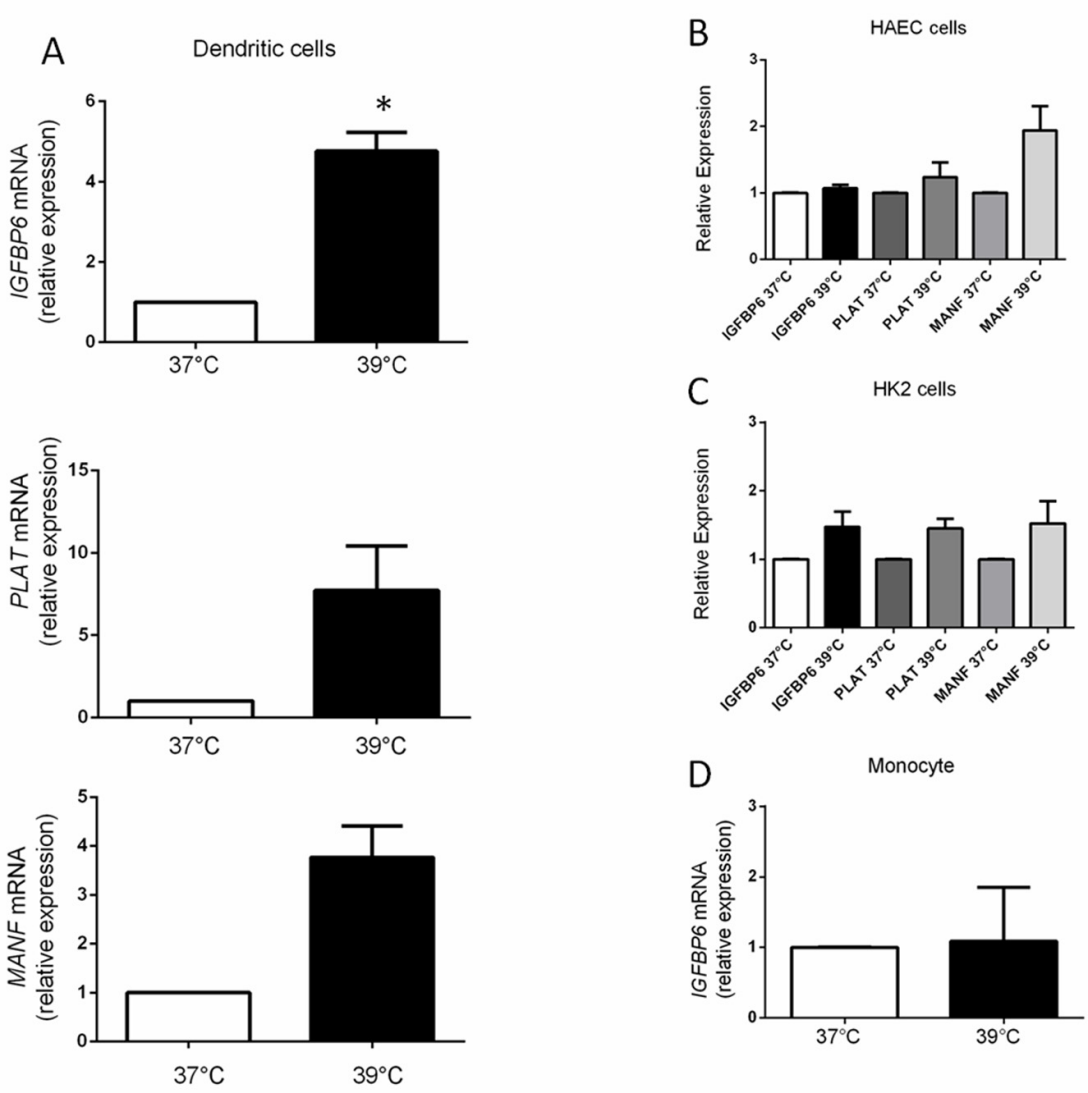
mRNA expression levels of *MANF*, *PLAT* and *IGFBP6* **A**. *MANF*, *PLAT* and *IGFBP6* expression by RT-PCR in DCs. mRNA levels were expressed relative to the *GAPDH* housekeeping gene by comparing PCR threshold cycle (CT) between cDNA of samples and *GAPDH* (ΔCt). Relative expression was obtained by computing the ∆∆Ct in comparison with the control sample (37°C) which was set at 1. Each bar represents mean ± SEM of four independent experiments each performed in triplicate. Statistical comparisons were made using paired data Student’s t-test. Differences were considered significant when *p* < 0.05. *IGFBP-6* was the only gene in which statistical significance could be demonstrated (*p = 0.004). **B.**, **C.** Expression levels of *MANF*, *PLAT* and *IGFBP6* by RT-PCR in HAEC and HK2 cells. In HAEC cells p-values for *IGFBP6, MANF*, *PLAT* were 0.26, 0.11, and 0.4 and in HK2 cells 0.16, 0.25, and 0.07 respectively. Each bar represents mean ± SEM of three independent experiments each performed in triplicate. Statistical comparisons were made using paired data Student’s t-test. **D.** Expression levels of *IGFBP6* by RT-PCR in monocytes exposed to hyperthermia. Monocytes do not upregulate IGFBP-6 in response to hyperthermia. Each bar represents mean ±SEM of three independent experiments each performed in triplicate. Statistical comparisons were made using paired data Student’s *t*-test.

We used quantitative RT-PCR to confirm upregulation of *MANF*, *PLAT* and *IGFBP6* in DCs from four normal individuals who were unrelated to the previous cohort. Surprisingly, we found that although overall upregulation of the three genes was observed (Figure 2A), *IGFBP6* was the only gene in which statistical significance could be demonstrated (*p* = 0.004), whereas *PLAT* (*p* = 0.089) and *MANF* (*p* = 0.05) did not reach statistical significance, probably due to variability in expression levels in a small cohort. These data show a selective up-regulation of *IGFBP6* in monocyte-derived DCs exposed to hyperthermia for only 3 h, suggesting *IGFBP6* as an early marker of fever-like temperature.

### *IGFBP6* is upregulated in DCs but not in other cell types exposed to hyperthermia

We then investigated whether hyperthermia-induced upregulation of the three genes (*IGFBP6, PLAT, MANF*) is a general biological phenomenon and whether any of those genes would be differentially regulated in DCs compared to other cell types exposed to 39°C. Accordingly, we compared expression as measured by quantitative RT-PCR in ontogenetically distant cell lines. In contrast to the ∼5-fold increased *IGFBP6* expression in DCs, there was no significant increase after hyperthermia in human aortic endothelial cells (HAEC), human renal cells (HK2), or a number of human cancer cell lines (Figure 2B and 2C, Supplementary Figure S2 in Supplementary Materials) [32,33]. *PLAT* and *MANF* expression were also not significantly upregulated in the two cell lines (HAEC and HK2) in which they were studied (Figure 2B and 2C).

We also evaluated whether monocytes *per se* upregulate *IGFBP6* expression in response to short exposure to hyperthermia, as they are directly related to DCs. We found that monocytes do not upregulate *IGFBP6* (*p* = 0.9) (Figure 2D). These data show that upregulation of *IGFBP6* by hyperthermia occurs specifically in DCs and suggest that the ability to upregulate *IGFBP6* is acquired at the dendritic stage, and therefore it might play a specific role in DC physiology. Given the consistency of *IGFBP6* results, we then decided to focus our efforts on *IGFBP6* in order to clarify whether it has additional properties and functions related to DC physiology.

### IGFBP-6 protein expression in DCs exposed to hyperthermia

**Figure 3:**
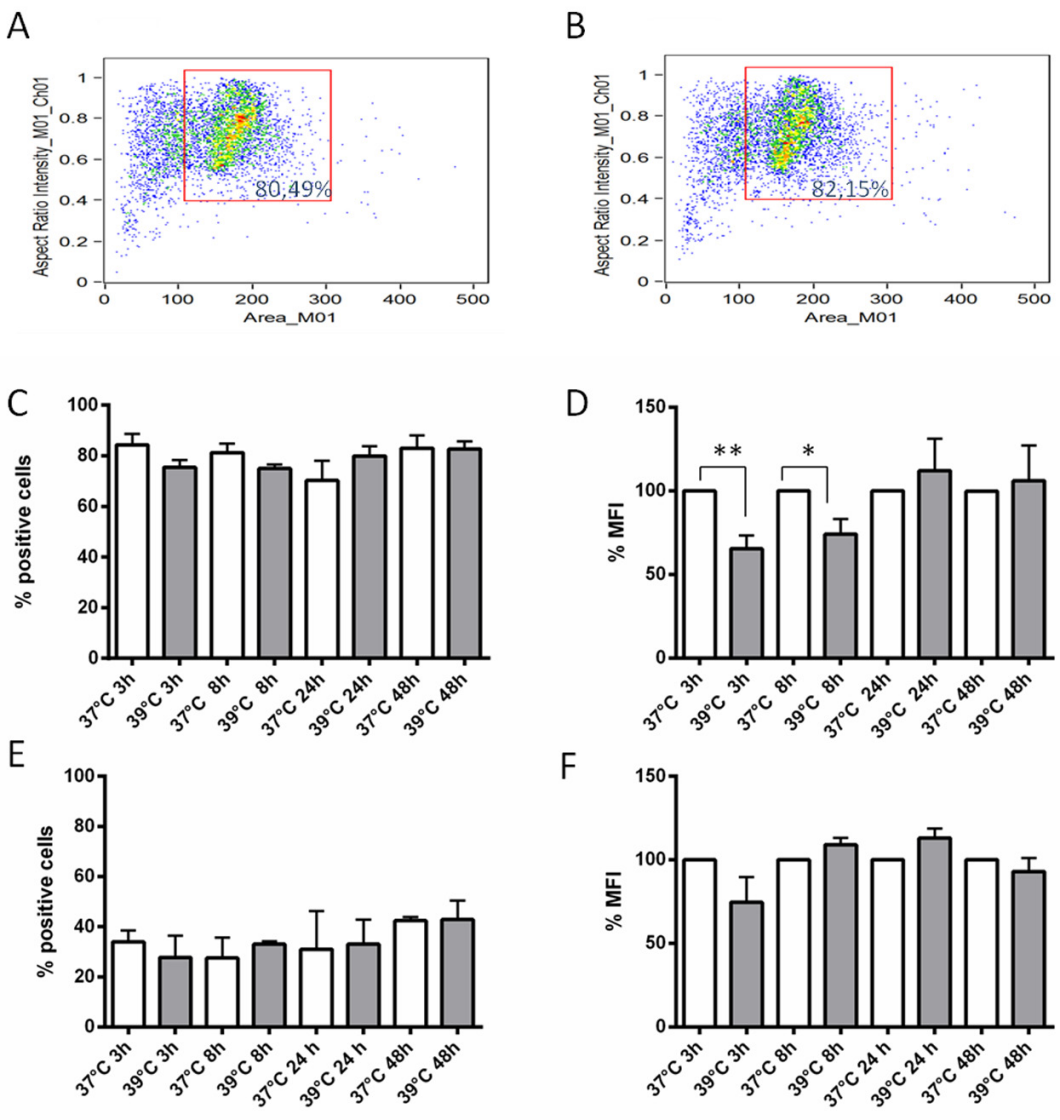
Expression levels of IGFBP-6 in DCs in presence or absence of permeabilization **A**., **B.** The dot plots, obtained by plotting the area of the cells (x-axis) *vs* the aspect ratio (*i.e.* the ratio between length and height) parameter (y-axis), displaying the typical distribution of DCs in presence (A) or in absence (B) of permeabilization, show that permeabilization did not alter cell viability. **C.**, **D.**, **E.**, **F.** The percentage of cells expressing IGFBP-6 after exposure at 39°C at different times and the relative percentage of mean fluorescence intensity (MFI) of cells exposed at 39°C towards cells exposed at 37°C (considered as 100%) were analyzed by flow cytometry in the presence (C and D, respectively) or absence of permeabilization (E and F, respectively). No staining was seen with controls using either secondary antibody alone or isotype control rabbit IgG polyclonal antiserum (not shown). Each bar represents mean ±SEM of four experiments. *p < 0.05; **p < 0.001. Statistical comparisons were made using unpaired Student’s t-test.

Since upregulation of the *IGFBP6* transcript was observed after a 3 h exposure to hyperthermia, we sought to investigate the time course of IGFBP-6 protein expression starting at 3 h. Flow cytometry with a specific monoclonal antibody revealed that DCs expressed detectable amounts of IGFBP-6 in comparison to samples stained with a control antibody both in permeabilized and non-permeabilized conditions (Supplementary Figure S3 and Supplementary Figure S4 in Supplementary Materials). Cell permeabilization was used to assess total protein expression. Cells were gated to exclude debris (Figure 3A), and the analysis was carried out on the gated populations. As shown in Figure 3C, ∼80% of permeabilized cells expressed IGFBP-6 irrespective of the temperature or exposure time. There was a significant decrease in the mean fluorescence intensity (MFI) of 39°C-exposed DCs at 3 and 8 h (Figure 3D), and this pattern was reflected by imaging of positive cells (Supplementary Figure S5 in Supplementary Materials). No significant change was observed in the percentages of IGFBP-6 positive cells at all time points (Figure 3C), indicating that the overall population remained positive for IGFBP-6 while decreasing its intracellular pool of the protein. In order to see whether IGFBP-6 is expressed on the plasma membrane, the same experiment was carried out in the absence of permeabilization. This experimental condition did not alter cell viability (compare Figure 3B with Figure 3A). The number of positive DCs was ∼50% lower than in the presence of permeabilization and did not change at 39°C (Figure 3E). Also, the MFI was not significantly affected by exposure to 39°C (Figure 3F). Individual cell imaging shows that IGFBP-6 was expressed on the plasma membrane and that the expression did not substantially change with temperature or time (Supplementary Figure S5 in Supplementary Materials). These data suggest that exposure to hyperthermia can decrease the pool of already synthesized IGFBP-6. We then hypothesized that IGFBP-6 was secreted upon exposure to 39°C. Thus, we investigated whether IGFBP-6 was detectable in conditioned media by immunoassay after 3, 8, 24 and 48 h of DC exposure to either 37°C or 39°C. IGFBP-6 concentrations were detectable only after 48 h of exposure at 39°C and found to be 0.635±0.023 ng/ml (*n* = 9), while the secreted protein was never detectable at 37°C.

**Figure 4:**
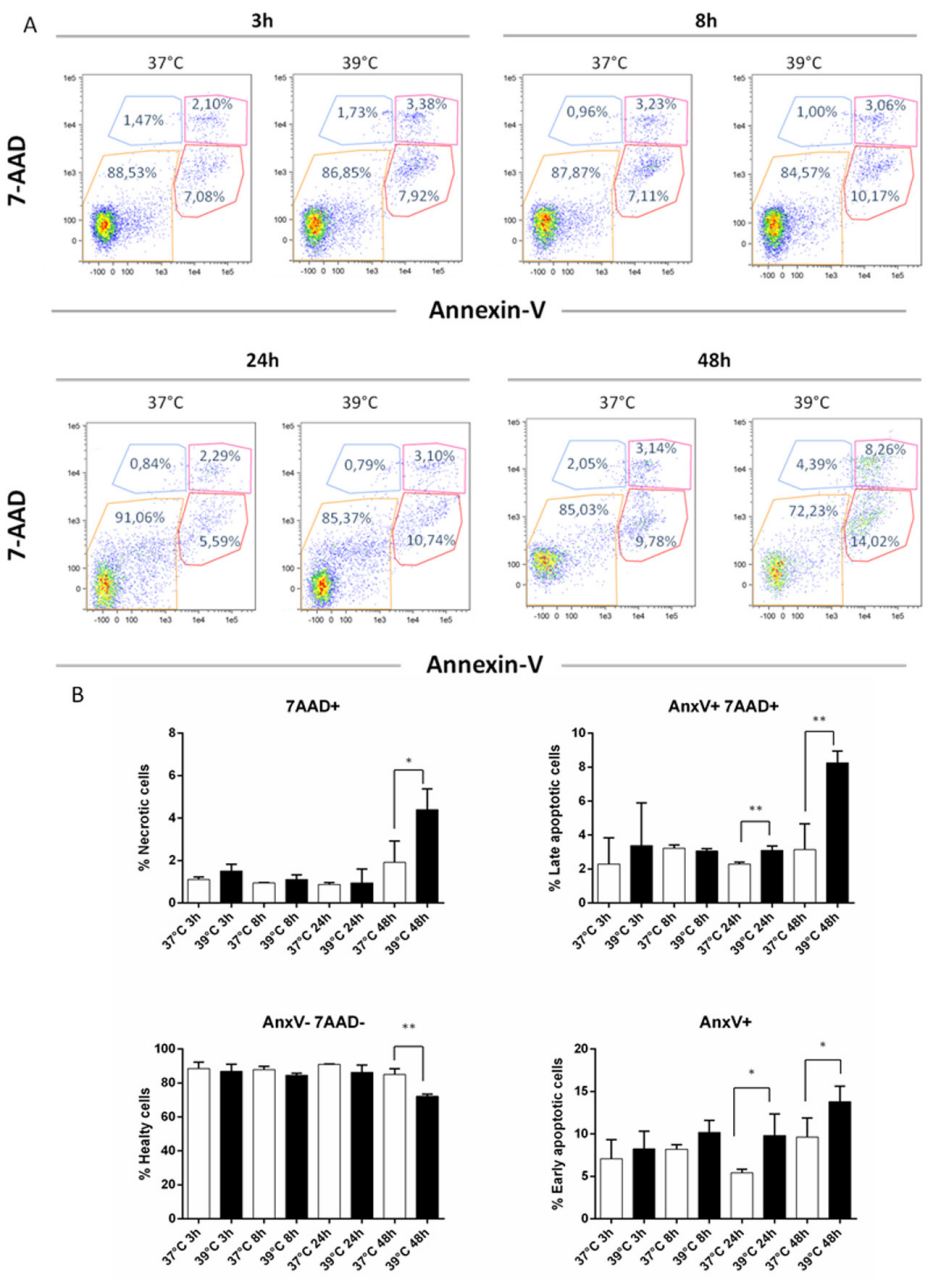
Apoptosis and necrosis induction by hyperthermia DCs were analyzed for necrosis/apoptosis levels after exposure to hypertermic conditions for different time points. **A.** Representative AnnexinV versus 7-AAD dot plots of DCs after 3, 8, 24 and 48 h of exposure to normal and hyperthermic conditions are shown. **B.** The percentages of healthy (Annexin V- and 7-AAD-), necrotic (Annexin V+ and 7-AAD+), early (Annexin V+ and 7-AAD-) and late apoptotic (Annexin V- and 7-AAD+) cells are shown as mean ± SEM of three independent experiments. *p < 0.05; **p < 0.001. Statistical comparisons were made using paired data Student’s t-test.

We then explored whether secretion was coupled to apoptosis and/or necrosis by incubating DCs for up to 48 h. As a positive control, DCs were incubated with H_2_O_2_ for 24 h (Table S1 in Supplementary Materials). As shown in Figure 4A, DCs displayed a different pattern of apoptosis and necrosis at 39°C compared to 37°C. As judged by the lack of staining with both 7-AAD and Annexin V, DCs viability was around 85% at all time-points at both 37°C and 39°C, with the notable exception of its decrease to 70% at 39°C for 48 h (Figure 4B). Concomitantly, late apoptosis and necrosis (AnxV-/7AAD+ and AnxV+/7AAD+) were both abruptly increased at 48 h, while early apoptosis (AnxV+/7AAD-) showed a more gradual increase at 39°C (Figure 4B). Overall, these data show that cell-associated IGFBP-6 protein expression decreases at early times upon exposure to hyperthermia and is followed by the appearance of IGFBP-6 in media coupled with apoptosis/necrosis of DCs at later times.

### IGFBP-6 promotes chemotaxis of monocytes and T lymphocytes

**Figure 5:**
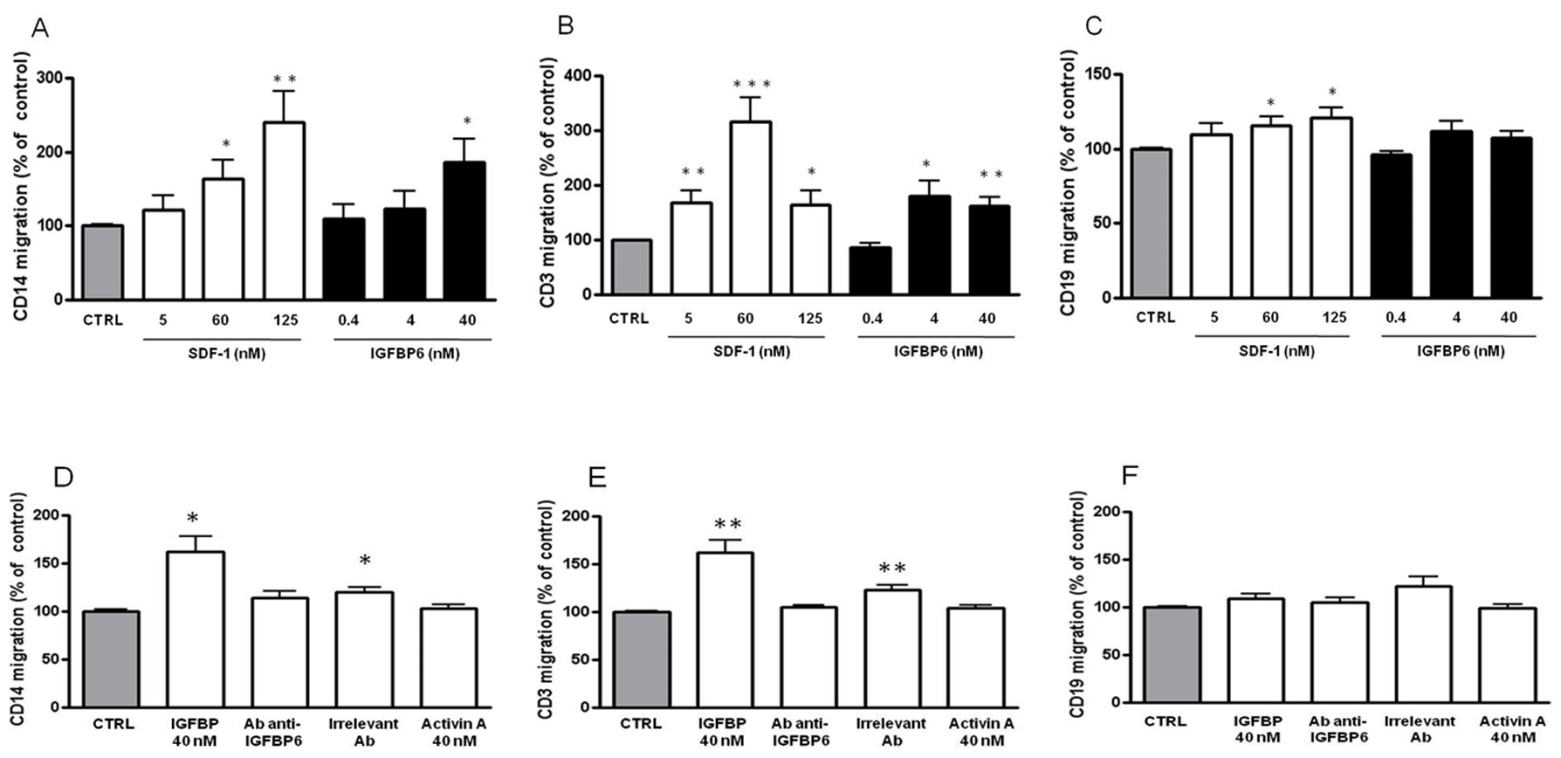
Chemoattractive effect of IGFBP-6 on monocytes, T lymphocytes and B lymphocytes **A.**, **B.**, **C.** Monocytes (A), T lymphocytes (B) and B lymphocytes (C) were placed in the upper compartment of a Transwell in the absence or presence of IGFBP-6 at different concentrations in the lower compartment. Number of migrating cells across a 3 µm-pore membrane after 150 min was measured by flow cytometry. As a positive control, experiments were conducted in the presence of different concentrations of SDF-1 in the lower compartment. Results are shown as mean ±SEM of three independent experiments and expressed as a percentage of controls obtained in the absence of IGFBP-6 in the basolateral compartment and set at 100%. *p < 0.05; **p < 0.001; ***p < 0.0001. **D.**, **E.**, **F.** In order to assess the specificity of the IGFBP-6 chemoattractive effect, monocytes (D), T lymphocytes (E) and B lymphocytes (F) were placed in the upper compartment of a Transwell in the presence in the lower compartment of: IGFBP-6 (40 nM); IGFBP-6 (40 nM) pre-incubated with an antibody directed against IGFBP-6; IGFBP-6 (40 nM) pre-incubated with an irrelevant antibody; Activin A (40 nM) as negative control. Results are shown as mean ±SEM of three independent experiments and expressed as a percentage of control. *p < 0.05; **p < 0.001. Results were analyzed using 2-way ANOVA with Tukey’s Multiple Comparison test.

Since we speculated that IGFBP-6 might have a novel role in immunity, based on its selective upregulation, we explored whether it could serve as a chemoattractant for monocytes and B and T lymphocytes using a Transwell migration assay. SDF-1, a known chemoattractant for these cell types [34-36], was used as a positive control. As shown in Figure 5A, supplementation of basolateral culture media with recombinant IGFBP-6 increased monocyte migration in a dose-dependent fashion to a maximum of 187±31% of control (*p* < 0.05). T cell chemotaxis was also significantly increased, showing a peak at 4 nM (0.1 µg/ml; 180±29 % of control, *p* < 0.05), with a behavior similar to SDF-1 (Figure 5B). Concentrations of IGFBP-6 lower than 4 nM had no chemotactic activity for T cells (Figure 5B). Notably, IGFBP-6 did not exert a significant chemotactic effect on B lymphocytes (Figure 5C). Preincubation of IGFBP-6 with an anti-IGFBP-6 antibody abolished the chemotactic activity, while preincubation with an irrelevant antibody unexpectedly had a partial effect, as demonstrated by the statistically significant difference with the controls (Figure 5D). Moreover, we observed that an irrelevant mouse protein (Activin-A) produced with the same system used for IGFBP-6 and SDF-1 expression (insect cell culture infected with baculovirus) had no chemotactic effect at the equivalent concentration (Figure 5D, 5E, and 5F).These data strongly indicate that IGFBP-6 has a chemotactic effect on T cells and monocytes but not on B cells.

## DISCUSSION

Fever is a fundamental host defense mechanism, which can occur in response to infectious agents, environmental stresses, medications and tumors. It is widely accepted that the physiological core body temperature is approximately 37°C and it is well conserved in vertebrates. Temperature is typically elevated to 38-40°C in a febrile response to infection or other stresses. It is important to note that *in vitro* experimental temperatures used across immunological studies performed in the past are rather heterogeneous [37]. Obviously, thermal stress in the range of common febrile responses can be considered more clinically relevant and therefore we chose to perform our gene expression profiling studies at 39°C.

It is becoming increasingly clear that different signals induce distinct programs of DC differentiation and different forms of immunity and tolerance [38]. In the past few years many advances have been made in addressing the action exerted by pathogen-associated molecular patterns (PAMPs), cytokines, chemokines, and other less characterized stress molecules on the activity of DCs. We analyzed the effects of temperature on gene expression programs in myeloid DCs generated *in vitro* from monocytes as a defined starting cell population. In fact, it has been clearly demonstrated that, in addition to functioning as macrophage precursors, monocytes have the capacity to differentiate into DCs, and therefore they play an essential role in both innate and adaptive immunity. In particular, both murine and human monocytes cultured with GM-CSF and IL-4 differentiate into immature DCs, characterized by their low expression of the costimulatory molecules CD80 and CD83 [39]. In keeping with our results (as shown in Supplementary Figure S1 in Supplementary Materials), these markers have been shown to be more highly expressed at 40°C [21]. Finally, CD11c has been found to be expressed at different levels in immature monocyte-derived DCs [40], and this is consistent with our data showing that about 50% of immature DCs are positive at 37°C, increasing to about 66% after 24 h at 39°C. Overall, we can conclude that mild thermal stress on DCs for 24 h at 39°C may enhance the function and immunostimulatory capacity of human DCs.

Obviously, *in vitro* monocyte differentiation systems cannot faithfully mimic all the physiological conditions that control *in vivo* monocyte differentiation. Furthermore, differentiation to DCs is generally observed in a fraction of the cultured monocytes, so this could also impair the detection of more subtle variation in gene expression. Nonetheless, these systems have proven to be a useful tool for studying factors that control DC differentiation, and, therefore, might provide information about the physiological situations in which this process takes place.

Four possible mechanisms have been proposed by which fever might confer protection [2-4]: i) directly killing or inhibiting growth of pathogens; ii) inducing cytoprotective HSPs in host cells; iii) inducing expression of pathogen HSPs, as activators of host defenses; iv) modifying and orchestrating host defenses. Our study highlights the central role of the heat shock response in the process of DC maturation upon fever-like conditions. Indeed, HSPs such as HSP60, HSP70 and HSP90 have been proposed to play a critical role in antigen presentation and DC activation [41] as part of their chaperone functions. It has been proposed that intracellular HSPs stimulate proteasome-dependent proteolysis and favor the generation of degradation products that are utilized as ligands of MHC class I molecules. This favors antigen recognition by CD8^+^ T cells, leading to activation of cytotoxic T cell immunity, which is indispensable in fighting viral infections [42]. In this context, HSP70 and HSP90 are involved in the cross-presentation of exogenous antigens by different antigen-presenting cells, including DCs and B cells. This is a direct consequence of their ability to act as chaperones and form complexes with peptides, which correlates with improved antigen delivery to endosomal compartments [43,44]. However, the putative role of HSPs in antigen cross-presentation has also been the subject of controversy [45]. Furthermore, several lines of evidence suggest that secreted extracellular HSP70 amplifies events associated with cytokine-dependent DC differentiation/maturation and may function to positively regulate the maturation of monocyte-derived DCs [46].

Since we explored global gene expression profiling in order to search for response genes besides HSPs involved in or governing the effects of hyperthermia on the immune system, it was intriguing that a few upregulated genes encoded secreted proteins, namely MANF, PLAT and IGFBP-6, which are potentially capable of interacting with other immune cells through receptors. Importantly, we showed that *IGFBP6* transcription is consistently and significantly upregulated by DCs as a response to hyperthermia. Also we show that *IGFBP6* upregulation is specific for DCs, as this gene is not upregulated in epithelial cell lines (of endothelial and renal origin) or monocytes. Rather, upregulation of the gene appears to be a distinct feature that DCs acquire when maturing from monocytes.

While we found up-regulated *IGFBP6* gene expression after 3 h of hyperthermia, we focused on the consequences of this increase at the protein level. Interestingly, intracellular staining did not reveal an increase in IGFBP-6 protein levels upon hyperthermia but rather a clear and significant decrease up to 8 hours with a later recovery (24-48 h), whereas membrane-bound staining was only slight decreased at 3 h. These data indicate that *IGFBP6* gene expression increase is not coupled with an increase in protein expression at earlier times and suggest that the decrease of intracellular IGFBP-6 could be recovered by subsequent synthesis. However, the decrease in cellular protein was not paralleled by the appearance of protein in the conditioned medium at earlier times, but rather required 48 h of hyperthermia. As many primary endogenous danger signals are released from the interior of the cell when the cell loses plasma membrane integrity upon necrosis [47], it is conceivable that necrotic DCs may also release signals able to activate adaptive immune cells. Therefore, we studied whether secretion of IGFBP-6 was linked to necrosis, finding actually that this was the case.

Notably, IGFBP-6 protein is well known to inhibit the actions of IGF-II including proliferation, differentiation, migration and survival in many cell lines [29]. However, more recent studies also suggest novel actions independent of IGF. In particular, IGFBP-6 (40 nM, 1 µg/ml) has been shown to induce chemotaxis in Rh30 rhabdomyosarcoma cells [30]. Therefore, we investigated whether IGFBP-6 possesses any chemotactic capability in regard to freshly isolated peripheral monocytes and lymphocytes. Importantly, we showed that IGFBP-6 induced chemotaxis of monocytes and T lymphocytes, but not of B lymphocytes. Interestingly, the chemotactic effect of IGFBP-6 was only seen at ∼150-fold higher concentrations than those found in the conditioned medium. Notwithstanding the problems with extrapolating i*n vitro* findings to the *in vivo* situation, this suggests that there may be a threshold of DC activation resulting in sufficient secretion of IGFBP-6 for it to mediate chemotaxis. Nevertheless, it can be speculated that secretion of the signal (IGFBP-6) from DCs during hyperthermia may ultimately favor DC-T cells encounters. However, currently we do not know if different DC subtypes (e.g. activated DCs) act differently in terms of their capacity to secrete IGFBP-6, nor if different T cell subsets *in vivo* would act differently when exposed to IGFBP-6. In fact, our results warrant further studies to shed light on what happens *in vivo*, with particular emphasis on lymphoid organs where T cells meet antigen and initiate an adaptive immune response. Given that IGFBP-6 has recently been associated with several pathological conditions [48-50], further studies are warranted in order to evaluate how IGFBP-6 is governing chemotaxis and whether this protein might also play a pathogenetic role in autoimmunity.

## MATERIALS AND METHODS

### Ethics statement

Specific approval of the local ethics committee was obtained for this study (Ospedali Riuniti University Hospital cod. 30/CE/2014). Written informed consent was obtained from all participants.

### Monocyte-derived dendritic cells isolation

Nine consecutive healthy adult blood donors were recruited without regard to age, ethnic origin, or gender. DCs were generated starting from selected CD14-positive cells obtained using Miltenyi MicroBeads (Miltenyi Biotec, Bergisch Gladbach, Germany). Briefly, after density gradient centrifugation, positively selected cells were plated in flasks or 75 cm^2^ roller bottles, at a density of 100 × 10^6^ cells in 10 ml of complete culture medium AIM-V (Invitrogen, Frederick, MD, USA) and incubated at 37°C and 5% CO_2_ adding GM-CSF (50 ng/ml) (Schering-Plough, Milan, Italy) and IL-4 (1000 U/ml) (Peprotech, London, UK). DCs were cultured for 6 days [23]. Finally, in order to confirm the expected DC phenotype, cells were stained with specific mAbs or appropriate isotype controls for 30 minutes at 4°C in FACS buffer Dulbecco’s PBS (Lonza, Basel, Switzerland) containing 2% FBS (PAA GmbH, Pashing, Austria), washed twice and finally resuspended in cold FACS buffer containing 0.1 µg/mL propidium iodide (PI) (Carl Roth, Karlsruhe, Germany). Stained cells were immediately analyzed with Epics XL-MCL™ flow cytometer (Beckman Coulter, Brea, CA, USA). Cell debris and dead cells were excluded from the analysis by gating on proper forward and sideward light scatter and on PI negative cells (∼98%). A minimum of 1 × 10^4^ living cells derived from a single flask per condition were analyzed for each sample and results were analyzed using Expo 32 ADC Software (Beckman-Coulter). The following monoclonal antibodies were used to determine the phenotype of DCs: anti-CD14; anti-CD80; anti-CD11c; anti-CD83; anti-HDRII (Beckman-Coulter). Some flasks were then moved to 39°C for 3 h or 24 h. In all cases, parallel control cultures were handled by culturing cells at 37°C.

### Gene expression profiles: sample preparation and hybridization

Cells were lysed in TRIZOL (Invitrogen, Frederick, MD, USA) and total RNA was further purified with the QIAGEN RNeasy kit following manufacturer’s instructions. RNA was quantified on the NanoDrop ND-1000 and quality checked with the 2100 Bioanalyzer (Agilent Technologies, Santa Clara, CA, USA). Five µg of total RNA were retrotranscribed from a T7-oligo(dT) primer with the SuperScript II polymerase (Invitrogen). cDNA was then purified on affinity columns and in vitro transcribed with the T7 RNA polymerase and a biotinylated dUTP. Labeled cRNA was purified on affinity columns and quantified on the NanoDrop ND-1000. Twenty µg of cRNA were fragmented and quality checked with the 2100 Bioanalyzer (Agilent Technologies).The biotinylated cRNA was hybridized to the Affimetrix HGU133 Plus 2.0 array, containing about 55,000 probe sets and open reading frames from the *H. sapiens* genome databases GenBank, dbEST and RefSeq. Chips were washed and scanned on the Affymetrix Complete GeneChip^®^ Instrument System, generating digitized image data (DAT) files.

### Microarray data analysis

DAT files were analyzed by Expression Console (Affymetrix Inc.). We used the Robust Multi alignment Algorithm [51] to normalize the full data set. The expression values obtained were analyzed by using GeneSpring GX 10 (Agilent Technologies). Further normalization steps included per-chip normalization to 50^th^ percentile and per-gene normalization to median. Normalized data were filtered for fold changes greater than 2, giving a list of 67 genes on 83 probe sets. We will refer to this set of differentially expressed genes as “focus gene list”. The fold change is defined as the ratio between the averages of the normalized expression values at temperatures of 39°C and at 37°C. Microarray data are available in the ArrayExpress database (www.ebi.ac.uk/arrayexpress) under accession number E-MTAB-697.

### Analysis of gene expression data

The analysis algorithm from Ingenuity Pathway Analysis (IPA; Ingenuity^®^ Systems, www.ingenuity.com) was used to identify the biological functions (IPA’s biofunction tool) and knowledge-based networks (IPA’s network generation tool) from the differentially expressed focus genes. The analysis points out the biological functions and/or diseases that were most significantly associated with the considered data sets, in particular leveraging information on the biological interactions stored in the Ingenuity Pathway Knowledge Base (IPKB). Focus genes were associated with biological functions and/or diseases in the IPKB. Fisher’s exact test [52] was used to calculate the probability (p-value) that each biological function/disease association is due to chance alone. Furthermore, resulting p-values were corrected for multiple testing using the Benjamini-Hockberg False Discovery Rate (FDR) [53].

### Cell lines

Human HK2 renal tubular cells were cultured in 50% DMEM and 50% HAM’s F12 supplemented with 10% fetal bovine serum, 2 mM L-glutamine, 100 IU/ml penicillin and 100 μg/ml streptomycin (Invitrogen). Human aortic endothelial cells (HAEC) were cultured in EBM™-2 (endothelial basal Medium-2), supplemented with EGM™-2 BulletKit™ (EBM™-2 plus SingleQuots™ of growth supplements) containing BBE (Bovine Brain Extract), hEGF, hydrocortisone, GA-1000 (Gentamicin, Amphotericin-B), FBS (Fetal Bovine Serum),VEGF, hFGF-B, R3-IGF-1, ascorbic acid, heparin (Lonza). Human colon cancer cell line (HCT116), human breast adenocarcinoma cell line (MCF-7 Cells), human prostate cancer cell line (PC3), heterogeneous human epithelial colorectal adenocarcinoma cell line (CACO2) were cultured in Dulbecco’s modified Eagle’s medium (DMEM) supplemented with 10% fetal bovine serum (FBS) , 1% PenStrep (10,000 U/mL Pen, 10 mg/mL Strep) 2 mM L-glutamine (medium and reagents from Sigma-Aldrich , Milan, Italy).

### Real Time RT-PCR analysis

For each sample, total RNA was isolated using TRIZOL (Invitrogen). RNA was quantified on NanoDrop ND-1000 and quality checked with the 2100 Bioanalyzer using the average A260/280 ratio of 2.0. For the first strand synthesis of cDNA, three µg of RNA were used in a 20 µl reaction mixture using Oligo (dT) primers and cDNA Superscript II (Invitrogen) according to the supplier’s instructions. For real-time PCR, the following primers were used: *IGFBP6*, forward 5’-GGAAGCTGAGGGCTGTCTC-3’, reverse 5’-GTCTCTGCGGTTCACATCCT-3’; *ARMET* forward 5’-CTGAGCACAGTGGACCT-3’, reverse 5’-GGCTGTTTTGGGAGTAA-3’; *PLAT* forward 5’-AGGGCTGGAGAGAAAAC-3’, reverse 5’-CTGGCTCCTCTTCTGAAT-3’; *GAPDH* forward 5’-CAAGGCTGAGAACGGGAA-3’, reverse 5’-GCATCGCCCCACTTGATTTT-3’. Primers were designed to be intron spanning.

The size of the products was also confirmed by gel electrophoresis for selected samples. mRNA levels were expressed relative to the housekeeping gene by comparing PCR threshold cycle (CT) between cDNA of samples and GAPDH (ΔCT). Relative gene expression subsequently was calculated as follows: fold change = 2^-*Δ(ΔCT)*^, where *∆CT = CTtarget - CThousekeeping* and *∆(∆CT)* = *∆CTtreated - ∆CTcontrol*. All experiments were performed in triplicate per sample. The Student’s t-test for paired data was used to assess gene expression at different temperatures for the three selected genes. All tests were two-sided and significance was set at *p* < 0.05.

### Flow cytometry

After incubation for 3, 8, 24 and 48 h at 39°C (or 37°C as control), cells were fixed in 3% paraformaldehyde and 2% sucrose in PBS and permeabilized (or not) with ice-cold Triton HEPES buffer (20 mM HEPES, 300 mM sucrose, 50 mM NaCl, 3 mM MgCl_2_, 0.5% Triton X-100, pH 7.4) for 5 min at room temperature. Then, cells were incubated with rabbit anti-human IGFBP-6 (Abcam; ab135606) for 1 h at 4°C (dilution 1:25), washed twice with PBS, and incubated with secondary antibody donkey anti-rabbit IgG (Alexafluor 488) for 30 minutes at 4°C (dilution 1:1000). As a negative control, cells were incubated either only with secondary antibody or with a rabbit IgG, polyclonal - isotype control (Abcam; ab37415) followed by incubation with secondary antibody. To evaluate the number of dead cells after fixation, Zombie Aqua Fixable Viability kit (BioLegend, San Diego, USA) was used. Then cells were washed twice with PBS, resuspended in 50 ml PBS and then analyzed by AmnisFlowsight IS100 (Merck Millipore). Brightfield scatter plots obtained by plotting Area (a parameter relative to cellular dimension) on x-axis vs Aspect Ratio (a parameter reflecting the ratio of the cell Minor Axis divided by the Major Axis) on y-axis were generated, then single cells events were gated, and finally 20,000 single-cell events for sample were acquired. The percentage of green positive cells (channel 2, 488 nm excitation laser) and mean fluorescence were analyzed using Amnis IDEAS software subtracting the values of the negative control. Brightfield and green fluorescent images for any single cell event were collected. A representative image of a single cell for any condition is shown. Zombie Dye staining revealed that an average of 4% of unfixed cells and 10% of fixed cells were dead.

### Analysis of secreted IGFBP-6 in conditioned medium

After 3, 8, 24 and 48 h exposure of DCs to either 37°C or 39°C, conditioned media were harvested and centrifuged at 200 × g at 4°C for 12 min and proteinase inhibitor (Aprotinin, Boehringer Mannheim GmbH) was added. Secreted protein in each sample was detected using a Bio-Plex cytokine, chemokine and growth factor assay (Bioclarma, Turin, Italy) according to manufacturer’s protocol. Briefly, the assay for IGFBP-6 was carried out in 96-well microplates using the Human IGF Binding Protein (IGFBP-6) Magnetic Bead Panel (HIGFBMAG-53K, Millipore) at the Bioclarma - Research and Molecular Diagnostics, Torino, Italy. Undiluted supernatant samples were treated according to manufacturer’s instructions and the contents of each well were drawn up into the Bio-Plex 100 System array reader (Bio-Rad), which identifies and quantifies each specific reaction based on bead color and fluorescent signal intensity. The data were finally processed using Bio-Plex Manager software (version 6.1) using five-parametric curve fitting and converted to ng/mL. The concentration of samples was obtained by comparing the fluorescence to that obtained from a standard curve. The sensitivity limit of the assay is 0.04 ng/mL.

### Apoptosis and necrosis

DCs were incubated for 3, 8, 24 and 48 h at 39°C (or maintained at 37°C as control) and evaluated for apoptosis/necrosis rate by using the FlowCellect^TM^ Annexin Red Kit (Merck Millipore), according to the manufacturer’s instructions. Briefly cells were stained with Annexin V conjugated with a sensitive dye CF647 (excitation laser: 642 nm, emission max: 670 nm) for 15 min at 37°C, washed in assay buffer, then stained with 7-AAD (excitation laser: 488 nm, emission max: 642 nm) for 5 min and analyzed by AmnisFlowsight IS100 (Merck Millipore). Brightfield aspect ratio versus brightfield area plots were generated to identify single cells events, then 20,000 single-cell events for sample were acquired. Dot plots were obtained by plotting the fluorescence of AnnexinV (channel 11) versus fluorescence of 7-AAD (channel 5), resulting in four different populations: (1) healthy cells, Annexin V(-) and 7-AAD(-); (2) necrotic cells, Annexin V(+) and 7-AAD(+); (3) early apoptotic cells, Annexin V(+) and 7-AAD(-); (4) late apoptotic cells, Annexin V(-) and 7-AAD(+).

### Cell chemotaxis

Human PBMC were isolated from buffy coats of healthy donors and used to purify CD14^+^ monocytes, CD19^+^ B cells and, CD3^+^ T cells with magnetic microbeads kit (Miltenyi Biotec). In order to evaluate chemotactic activity of IGFBP-6, monocytes, T and B lymphocytes were added (10^6^ per filter) to the upper compartment of 0.33 cm^2^ Transwells with 3 µm-pore filters (Corning, Acton, MA, USA), while no IGFBP-6 (as control) or different concentrations of IGFBP-6 (Peprotech, London, UK) (0.01 µg/ml, 0.1 µg/ml, 1 µg/ml) corresponding respectively to 0.4, 4, and 40 nM were dissolved in serum free Eagle’s Medium minimal essential medium (MEM; Sigma-Aldrich, Milan, Italy) in the lower compartment. As a positive control, different concentrations of SDF-1 (Peprotech, London, UK) (0.05 µg/ml, 0.5 µg/ml, 1 µg/ml) corresponding respectively to 6 nM, 60 nM and 125 nM were added to the lower compartment. In order to assess for the specificity of the IGFBP-6 chemotactic effect, we pre-incubated IGFBP-6 at the highest dose with a rabbit IgG anti-human C-terminal IGFBP-6 antibody (Abcam, Cambridge, UK) or with an irrelevant rabbit IgG antibody (anti-human HVCN1, Abcam, Cambridge, UK) in a weight ratio (IGFBP-6/antibody) of 1:6 for 30 min at room temperature, before adding the mixture in the lower compartment. Moreover, as a negative control, we used recombinant human/murine/rat (insect-derived) Activin A (Peprotech, London, UK), in the lower compartment at a dose of 40 nM. After 150 min, cells were recovered in the lower compartment and quantified by flow cytometry, and data were expressed as percentage of migration of the control (100%). The concentrations used for SDF-1 were previously shown to be optimal for chemotaxis of monocytes, T and B lymphocytes [29-31].

### Statistical methods

Data obtained from apoptosis/necrosis and chemotaxis experiments are shown as mean ± standard error of the mean (SEM) and analyzed for statistical significance using 2-way ANOVA with Tukey’s Multiple Comparison test using Graphpad Software v. 4 (La Jolla, CA, USA). *p* < 0.05 was considered statistically significant.

## SUPPLEMENTARY MATERIALS FIGURES AND TABLE


