# Supplementary material for: Human monocyte-derived dendritic cells exposed to hyperthermia show a distinct gene expression profile and selective upregulation of IGFBP6
Source: Oncotarget. 2017 Jun 1;8(37):60826–40. doi: 10.18632/oncotarget.18338 (PMC5617388; doi:10.18632/oncotarget.18338)
Supplement: Supplementary file 1 [file oncotarget-08-60826-s001.pdf]

Human monocyte-derived dendritic cells exposed to hyperthermia show a distinct gene expression profile and selective upregulation of *IGFBP6*

Supplementary Material

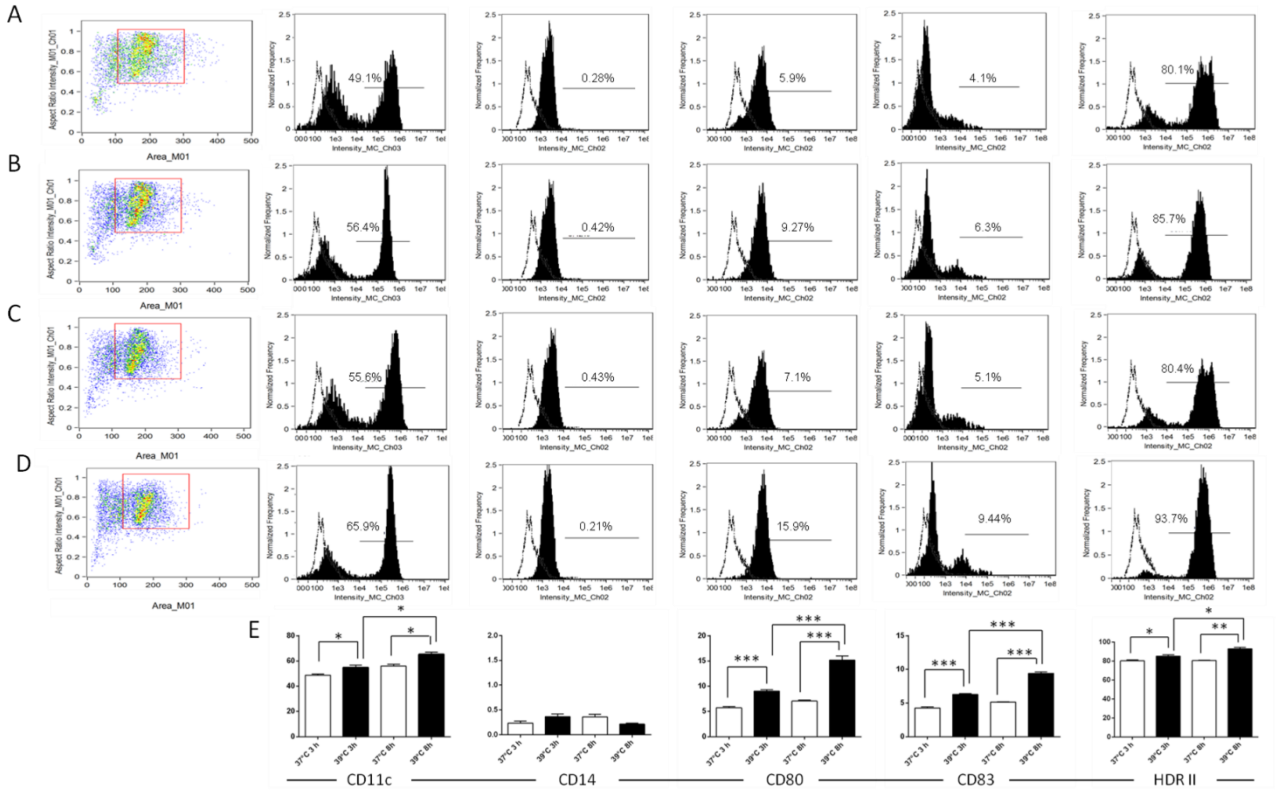

Supplementary Figure 1. Phenotypic analysis of dendritic cells. Characterization of dendritic cells (DCs) was performed at day six of differentiation after incubation for 3 h at 37°C (**A**) and 39°C (**B**) and for 24 h at 37°C (**C**) and 39°C (**D**) by labelling them with specific antibodies (black filled histograms). Unstained controls are shown as dotted lines. Gating strategy: representative scatter plots obtained by plotting Area (x-axis) vs Aspect Ratio (y-axis) for each condition, and gating single cells, are shown in the first column. The panels are representative of one case of the nine analyzed for gene expression. **E**. The percentage of maturation markers after exposure at 39°C and 37°C at different times. Each bar represents mean ± SEM of four independent experiments. Statistical comparisons were made using the unpaired Student's t-test. *p<0.05; **p<0.001; ***p<0.0001.

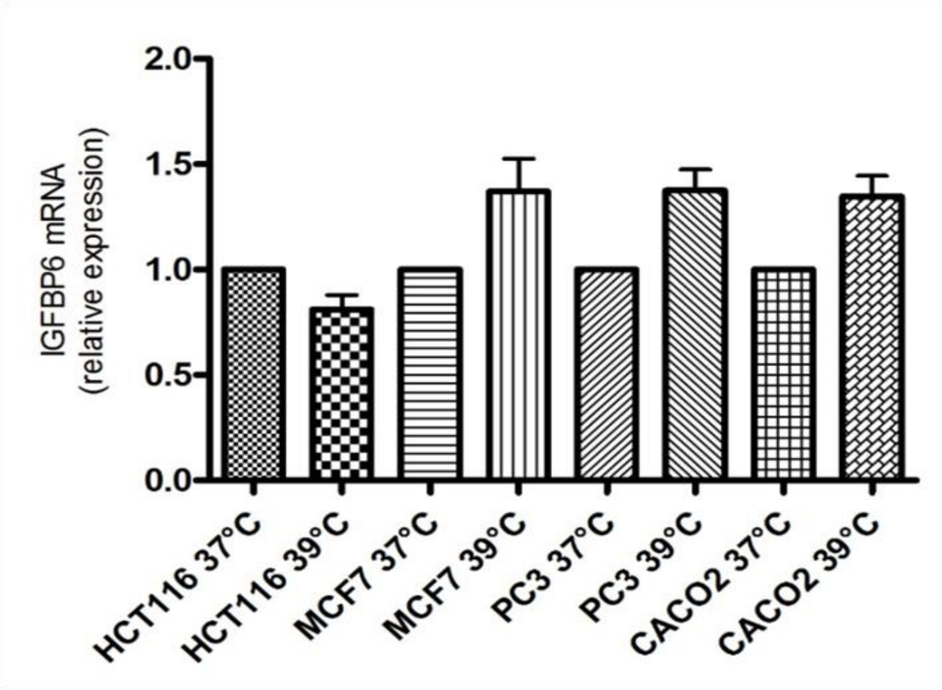

Supplementary Figure 2. Expression levels of *IGFBP6* by RT-PCR in HCT116, MCF7, PC3 and CACO2 cells. Relative expression levels of *IGFBP6* by RT-PCR after 3 hours of hyperthermia in HCT116, MCF7, PC3 and CACO2 cells compared to the same cell lines at 37°C. Each bar represents mean ± SEM of three independent experiments.

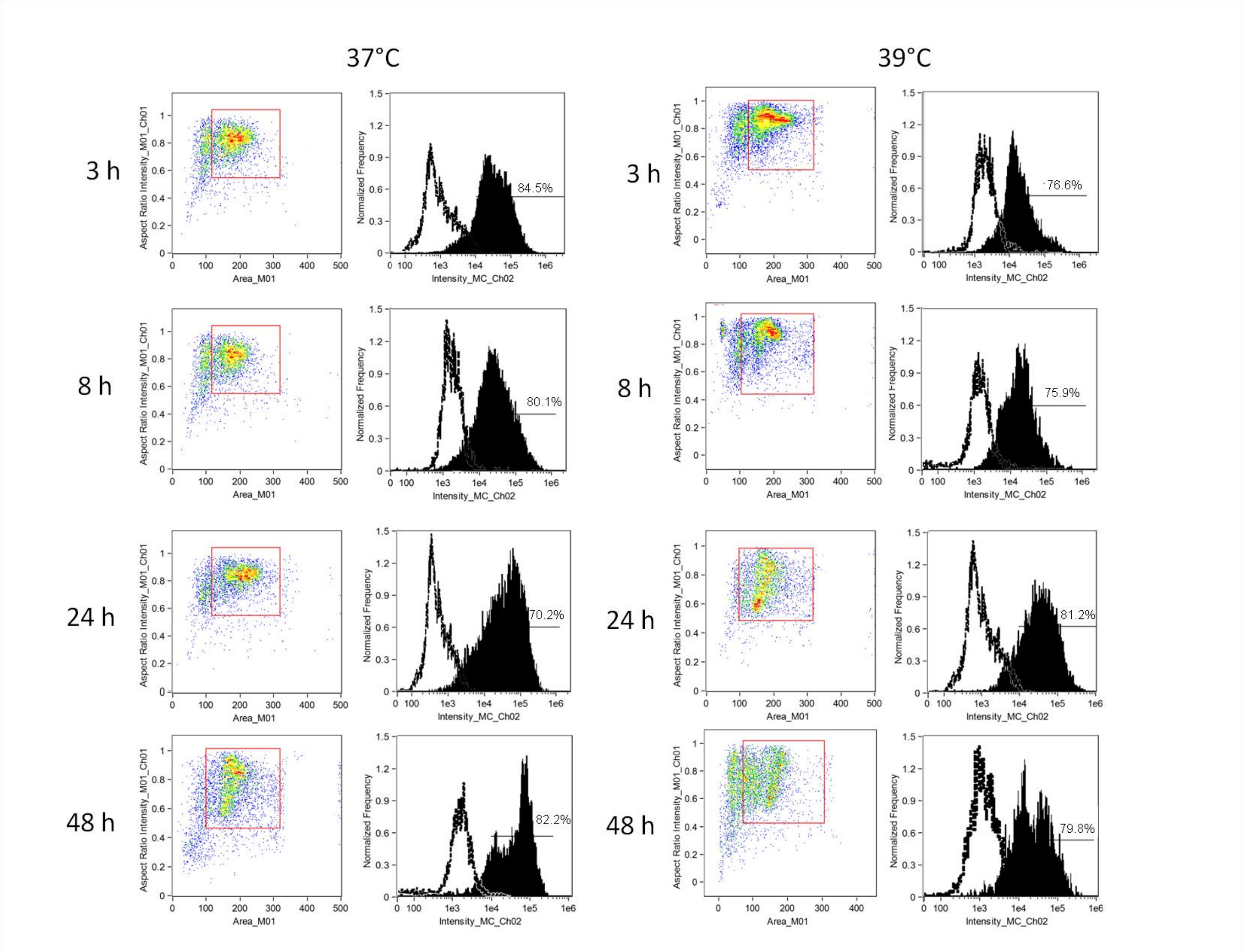

Supplementary Figure 3. IGFBP-6 expression in permeabilized dendritic cells. IGFBP-6 expression was analyzed in dendritic cells (DCs) at 37°C or after exposure at 39°C for different times. DCs were permeabilized and labelled with an antibody directed against IGFBP-6, followed by a secondary FITC antibody (black filled histograms). Histograms relative to cells stained only with secondary antibody (controls) are shown with dotted lines. Gating strategy: scatter plots of control DCs at 37°C or 39°C obtained by plotting the Area vs Aspect Ratio, and single cell gating, are shown in the first and third columns. The panels are representative of one case of the nine analyzed for gene expression.

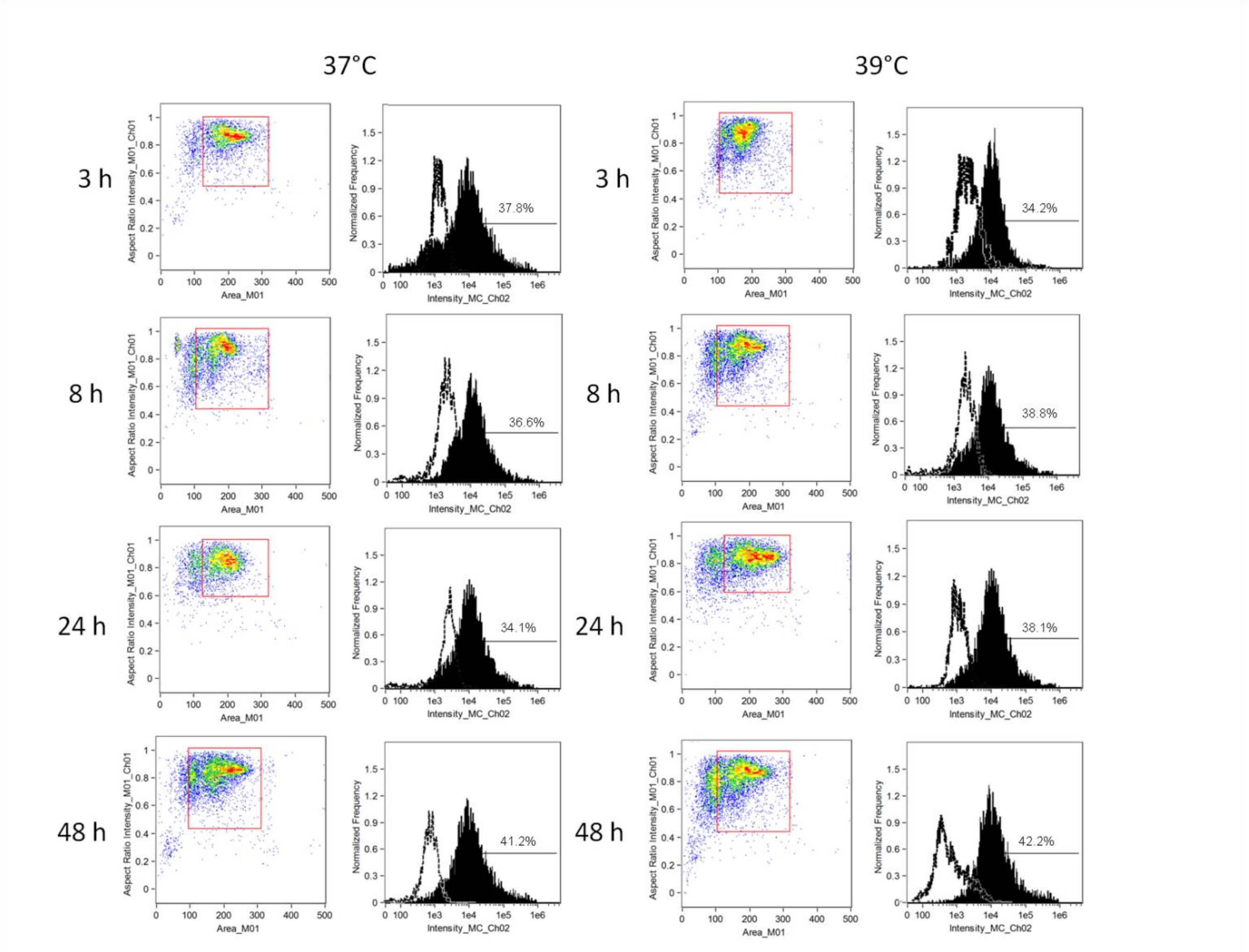

Supplementary Figure 4. IGFBP-6 expression in non-permeabilized dendritic cells. IGFBP-6 expression was analyzed in dendritic cells at 37°C or after exposure at 39°C for different times. DCs were labelled with an antibody directed against IGFBP-6, followed by a secondary FITC antibody (black filled histograms). Histograms relative to cells stained only with secondary antibody (controls) are shown with dotted lines. Gating strategy: scatter plots of control DCs at 37°C or 39°C obtained by plotting the Area vs Aspect Ratio, and single cell gating, are shown in the first and third columns. The panels are representative of one case of the nine analyzed for gene expression.

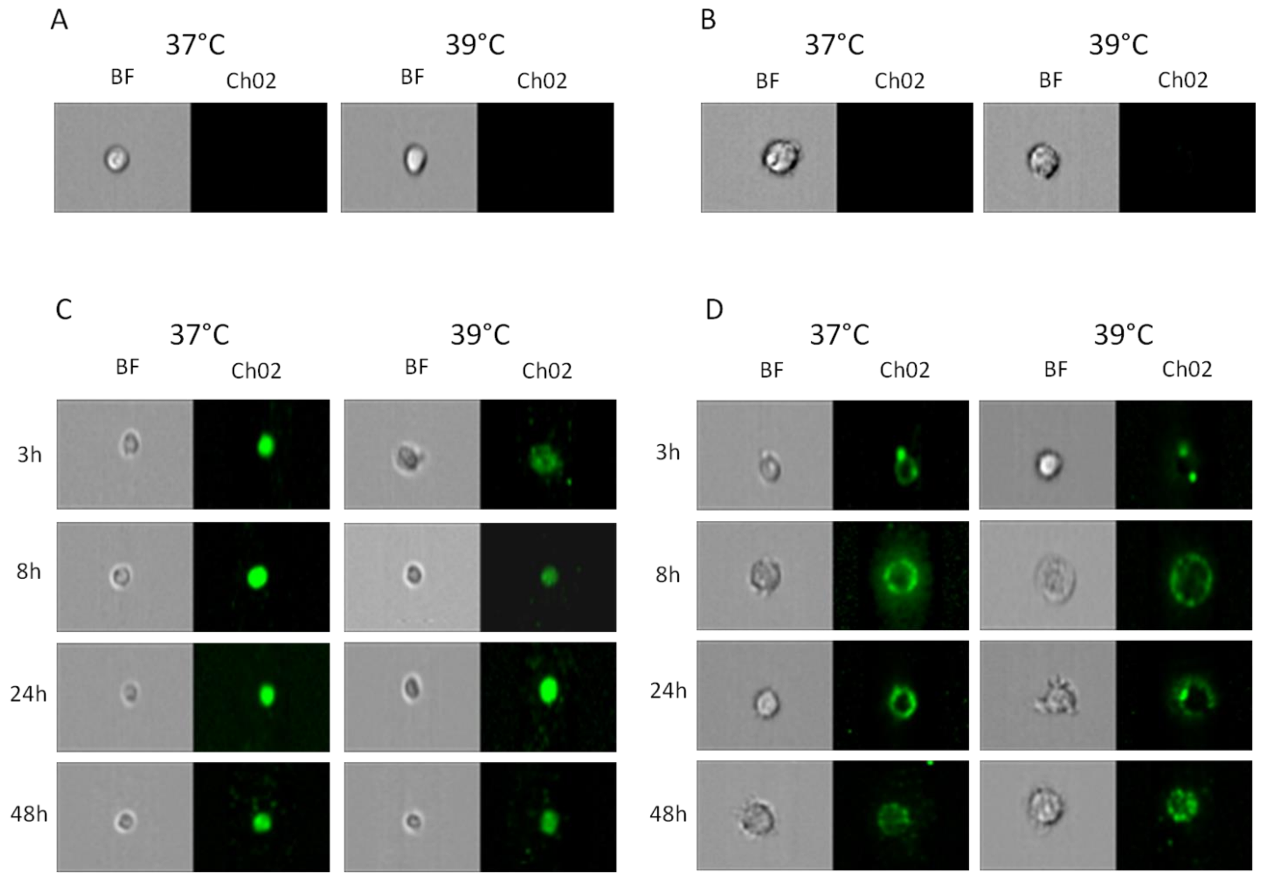

Supplementary Figure 5. Expression of IGFBP-6 in DCs in the presence or in the absence of permeabilization. Brightfield (BF) and green fluorescent images (Ch02) of representative single cells are shown. Negative controls were incubated only with FITC-conjugated secondary antibody in the presence (**A**) or in the absence (**B**) of permeabilization. **C, D**. DCs incubated with the anti-IGFBP-6 primary antibody and then with the secondary antibody in the presence (**C**) or in the absence of permeabilization (**D**).

Supplementary Table 1. Effects of H_2O_2 on DC viability.

	Cell labeling (%)			
	$AnxV^-$ $7\text{-}AAD^-$	$AnxV^+$	$AnxV^+$ $7\text{-}AAD^+$	$7\text{-}AAD^+$
37°C	86.4±0.9	8.24±1.02	3.02±0.92	1.25±0.22
37°C+H_2O_2	70.0±0.05	17.35±0.55	6.60±0.20	6.04±0.05

DCs (5 x 10^5) were incubated in medium alone or with H_2O_2 100 μM for 24 h and then evaluated by flow cytometry. Annexin V^+ staining corresponds to early apoptosis, Annexin V^+ $7\text{-}AAD^+$ staining to necrotic cells, $7\text{-}AAD^+$ staining correspond to late apoptosis. Data represent mean ± SEM of three independent experiments.
